# Interaction webs in arctic ecosystems: Determinants of arctic change?

**DOI:** 10.1007/s13280-016-0862-x

**Published:** 2017-01-23

**Authors:** Niels M. Schmidt, Bess Hardwick, Olivier Gilg, Toke T. Høye, Paul Henning Krogh, Hans Meltofte, Anders Michelsen, Jesper B. Mosbacher, Katrine Raundrup, Jeroen Reneerkens, Lærke Stewart, Helena Wirta, Tomas Roslin

**Affiliations:** 1 Department of Bioscience, Arctic Research Centre, Aarhus University, Frederiksborgvej 399, 4000 Roskilde, Denmark; 2 Department of Agricultural Sciences, University of Helsinki, P.O.Box 27, 00014 Helsinki, Finland; 3 GREA, 16 rue de Vernot, 21440 Francheville, France; 4 Department of Bioscience, Arctic Research Centre, Aarhus University, Grenåvej 14, 8410 Rønde, Denmark; 5 Department of Bioscience, Soil Fauna Ecology and Ecotoxicology and Arctic Research Centre, Aarhus University, Vejlsøvej 25, 8600 Silkeborg, Denmark; 6 Department of Bioscience, Aarhus University, Frederiksborgvej 399, 4000 Roskilde, Denmark; 7 Department of Biology, University of Copenhagen, Universitetsparken 15, 2100 Copenhagen, Denmark; 8 Greenland Institute of Natural Resources, Kivioq 2, P.O. Box 570, 3900 Nuuk, Greenland; 9 Animal Ecology Group, University of Groningen, Nijenborgh 7, 9747 AG Groningen, The Netherlands; 10 Department of Ecology, Swedish University of Agricultural Sciences, P.O. Box 7044, 750 07 Uppsala, Sweden

**Keywords:** Ecosystem function, Ecosystem services, Network, Plant–herbivore, Plant–pollinator, Predator–prey

## Abstract

How species interact modulate their dynamics, their response to environmental change, and ultimately the functioning and stability of entire communities. Work conducted at Zackenberg, Northeast Greenland, has changed our view on how networks of arctic biotic interactions are structured, how they vary in time, and how they are changing with current environmental change: firstly, the high arctic interaction webs are much more complex than previously envisaged, and with a structure mainly dictated by its arthropod component. Secondly, the dynamics of species within these webs reflect changes in environmental conditions. Thirdly, biotic interactions within a trophic level may affect other trophic levels, in some cases ultimately affecting land–atmosphere feedbacks. Finally, differential responses to environmental change may decouple interacting species. These insights form Zackenberg emphasize that the combination of long-term, ecosystem-based monitoring, and targeted research projects offers the most fruitful basis for understanding and predicting the future of arctic ecosystems.

## Introduction

All living organisms are embedded in interaction webs: individuals interact within and among populations, and these interactions play important roles in shaping the structure (i.e. who interacts with whom and how strongly) and ultimately the dynamics of ecosystems (sensu Hooper et al. 2005; Legagneux et al. 2014). How species are tied together in this web of interactions has been shown to affect the stability of populations and communities, and may affect the way species respond to environmental change (Tylianakis et al. 2008). Importantly, the structure of mutualistic and antagonistic interactions may affect overall dynamics in different ways, with a higher connectance usually increasing stability for mutualistic interactions, but decreasing it for antagonistic interactions (Thébault and Fontaine 2010). Even weak interactions may have a strong impact on the overall stability of the system (McCann et al. 1998). In fact, changes in environmental conditions may change the structure of the interaction web and the strength of biotic interactions—even in the absence of changes in more traditional ecological metrics such as species richness or community composition (Memmott et al. 2007; Tylianakis et al. 2008).

In the Arctic and elsewhere, some of the most conspicuous antagonistic interactions are herbivory and predation. Both herbivores and predators have direct impacts on the individuals that they forage or prey upon. However, the effects of herbivory and predation may extend beyond these direct interactions, for instance by altering the competitive interactions among individuals or species (Virtanen 1998; Olofsson et al. 2002), by decreasing the abundance of the preferred forage plants (Virtanen et al. 1997; Olofsson et al. 2002; Bråthen et al. 2007) or prey species (Gilg et al. 2003; Schmidt et al. 2008). This may, in turn, alter the diversity and ultimately the structure and functioning of entire ecosystems.

Among mutualistic interactions, the interaction between flowering plants and their pollinators may be one of the most important ecological interactions in nature (Hegland et al. 2009; Bascompte and Jordano 2013). Other important mutualistic interactions include the dispersal of plant seeds by animals (Bruun et al. 2008; Bascompte and Jordano 2013), and the reallocation of nutrients through consumption and excretion (Elton 1927; Mosbacher et al. 2016). Within this continuum ranging from exploitation to mutual benefit, there are numerous examples of other types of biotic interactions. Since these are the processes that tie together the web of interacting species in ecosystems, the biotic interactions have the capacity to convey influences from one compartment or process in the interaction web onto adjacent ones. Influences may thus cascade through the entire interaction web through biotic interactions.

In this paper, we synthesize our current knowledge about the structural and functional complexity of biotic interactions in Greenland, drawing on the rich monitoring and research efforts conducted over the past two decades within the Greenland Ecosystem Monitoring programme. We scrutinize the structural complexity of the high arctic ecosystems, and aim at deciphering and mapping the interaction web at Zackenberg. Through presentation of selected key interactions, we will shed light on the various aspects of such biotic interactions and assess their implications in the context of environmental change.

## A brief history of Arctic interaction webs

Given the central role of biotic interactions for the functioning of ecosystems, knowledge about how interaction webs are structured in the far North is crucial for understanding the consequences of ongoing and future climate change. In the Arctic, low temperatures, short growing seasons, and limited availability of nitrogen have created some of the least productive and species-poor ecosystems in the world (Nadelhoffer et al. 1991; CAFF 2013). Historically, the low species diversity in the Arctic (Willig et al. 2003; Jenkins et al. 2013) has led to the assumption that the interaction webs of the Arctic are simple too (e.g. Post et al. 2009; Legagneux et al. 2012).

**Fig. 1:**
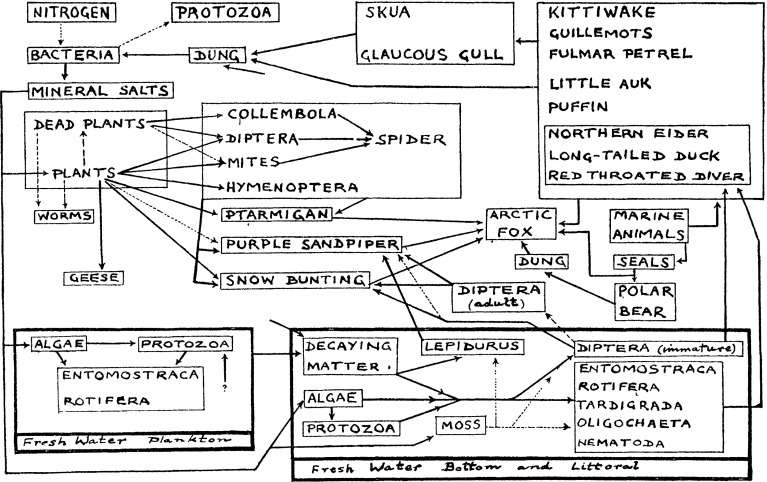
The original view on arctic food webs: a simple construction of few taxa connected by sparse interactions. Note the preponderance of vertebrate taxa, and the pooling of species-rich taxa into summary groups such as “Diptera” or “plants”. Reprinted with permission from Summerhayes and Elton (1923): Bear Island, Journal of Ecology 11:216–33, by Wiley, and the British Ecological Society

Understanding “who eats whom” in these remote regions has been part of arctic exploration from the earliest expeditions to Northeast Greenland. Already on Nordenskiöld’s Vega expedition in the nineteenth century, zoologist Stuxberg dissected animals to find out what they ate. These sporadic forays into the diets of individual species were first united by the father of modern animal ecology, Charles Elton. An Oxford University expedition to the remote high arctic location of Bjørnøya (Bear Island, south of Spitsbergen) yielded the first “modern” food web (Fig. 1). This description of the “Nitrogen Cycle” has remained highly influential in terms of how arctic interaction webs are thought to be structured. Based on the few species and the low number of trophic links depicted by Summerhayes and Elton (1923), arctic food webs have been considered generally simple constructs, low on species, and poorly connected.

Importantly, the structure of the webs constructed by Summerhayes and Elton (1923) was as much determined by what it does not show as by what it shows (Hodkinson and Coulson 2004). Being classic vertebrate zoologists, Summerhayes and Elton listed some twenty species of birds, a few mammal species, whereas invertebrate species and plant species were assigned to summary bins (Fig. 1). This view of the web prevailed for almost a century, until Hodkinson and Coulson (2004) revisited the original description of the food web of Bjørnøya. They stressed that the web consisted of many more species than previously revealed, and that the main part of diversity was hidden in the nodes left unresolved in previous webs.

**Fig. 2:**
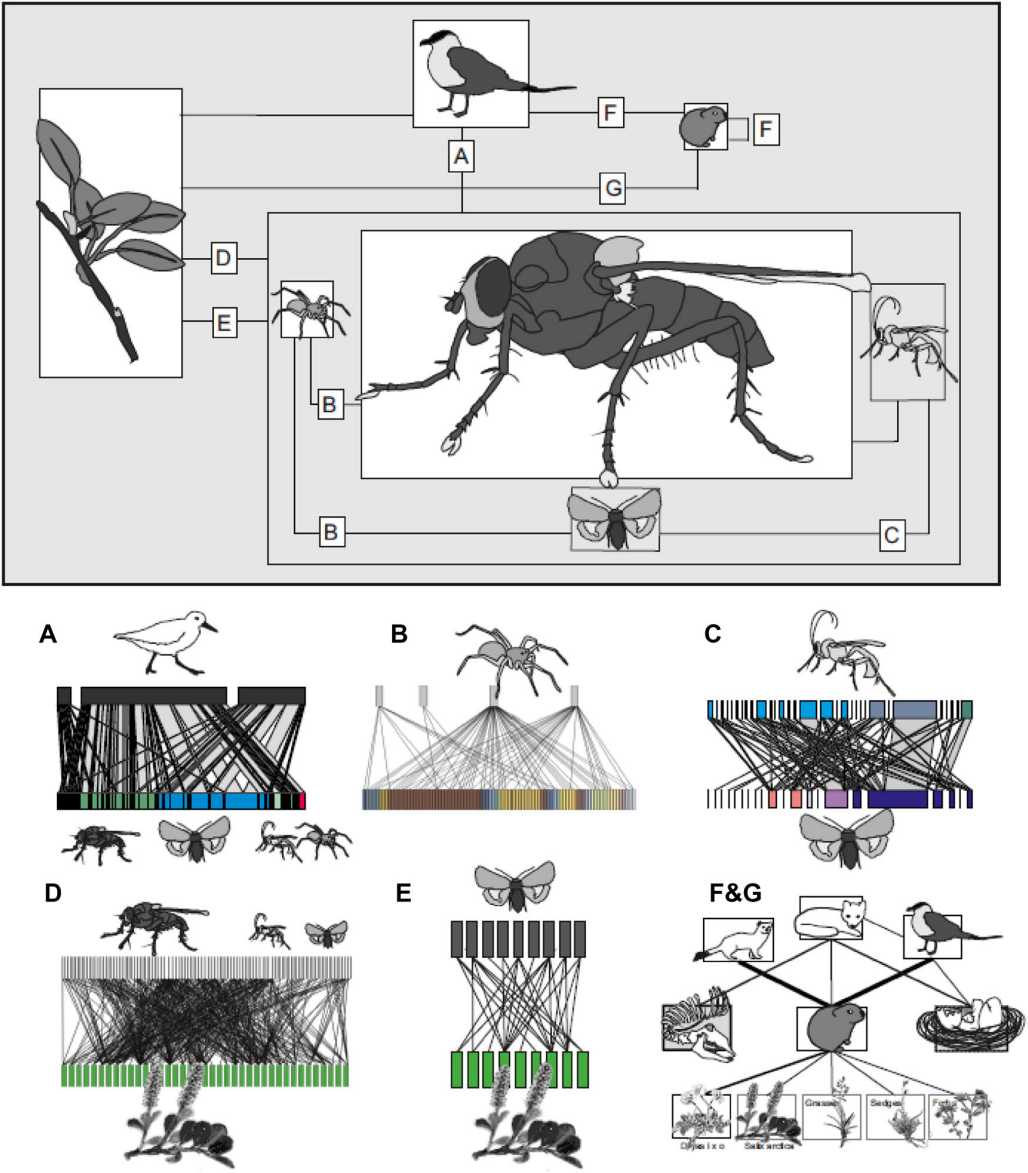
The various players of the interaction web at Zackenberg, as resolved by 20 years of interaction studies. In the *upper panel*, the species richness of each taxon is represented by the size of each *box*. Shown below the compound panel are selected interactions among specific guilds, as resolved by multiple studies. In all panels **a**–**f**, the *blocks* (irrespective width) represent one species at a trophic level. A *line* connecting the two levels represents an ecological interaction empirically detected. All interactions but **d** are antagonistic in nature. The specific interaction types represented are as follows: **a** birds and arthropod prey. Note that this graph is only semi-quantitative, showing the abundances of interactions and prey but not of birds. From Wirta et al. (2015a); **b** spiders and Dipteran and Lepidopteran prey. Note that this graph is qualitative and hence includes no information on the frequency of taxa or the interactions between them. From Wirta et al. (2015a); **c** Lepidoptera and their parasitoids. Shown is the consensus web emerging from a combination of three methods, MAPL-HL, MAPL-AP, and rearing. Here, the *boxes* and the *lines* connecting them only reflect the number of individuals involved in each interaction, whereas no data on the specific abundances of hosts and parasitoids are provided. *Colours* identify families. From Wirta et al. (2014); **d** plants and their pollinators. Note that this graph is qualitative and hence includes no information on frequency of taxa or the interactions between them. From Rasmussen et al. (2013); **e** plants and their invertebrate herbivores. Note that this graph is qualitative and hence includes no information on frequency of taxa or the interactions between them. From Roslin et al. (2013); **f** vertebrate predators and vertebrate prey in the lemming–predator system and **g** plants and vertebrate herbivore in the plant–lemming system. In these two plots, the strength of the connectors is proportional to frequency in lemming diet, and to the semi-quantified dietary fraction in predators. Muskox carcasses and eggs of ground-nesting birds represent alternative prey. Based on Schmidt et al. (2008) and Ehrich et al. (2015). All data are available upon request from the authors

Work conducted at Zackenberg, Northeast Greenland, has further upset previous descriptions of the presumed simplicity of arctic interaction webs. By sampling the local flora and fauna by a range of techniques (Olesen et al. 2008; Rasmussen et al. 2013; Wirta et al. 2014, 2015a, 2016), we have been able to map out the main part of macroscopic animal species and vascular plants. By then constructing molecular tools for identifying all local species (Wirta et al. 2016), we have been able to work out the general blueprint of the interaction web. Some parts of the interaction web are currently known with high and others with low precision, but the many links identified among the species currently known from Zackenberg yields substantial complexity (Fig. 2). Taking off from this rough sketch of this high arctic interaction web, we use the next sections to highlight central aspects of biotic interactions as revealed by research during the past two decades.

## Arctic webs are more complex than anticipated and dominated by arthropod species

After twenty years of dissecting the Zackenberg interaction web, four major insights have emerged: first, the interaction web is numerically dominated by arthropods (Roslin et al. 2013; Várkonyi and Roslin 2013; Wirta et al. 2015a, b, 2016). Second, which methods you use to resolve the web will affect the perception of the web (Wirta et al. 2014). Third, the structure of the web is far more complex than previously thought (Wirta et al. 2015a). Fourth, the structure of the web is highly variable in space and time (Rasmussen et al. 2013; Wirta et al. 2016). Each of these insights comes with major implications for how we should understand arctic communities and ecosystems and how they might respond to change.

As in most other places on Earth, the terrestrial interaction web at Zackenberg is numerically dominated by arthropod species. This can be demonstrated by some simple statistics: overall, 403 terrestrial animal species are currently known from Zackenberg. Of these, 336 are arthropod species, whereas only 67 are vertebrate species (60 birds, including rare visitors, and 7 mammals including the polar bear *Ursus maritimus*; Wirta et al. 2016). Importantly, the occurrence of vertebrates is registered in detail, whereas the arthropods are substantially under-sampled. Yet, the diversity of for instance midges (Chironomidae) apparently outnumbers mammals by a factor of at least 10:1 and more likely 20:1, thereby exceeding even tropical Diptera-to-Mammals ratios (cf. Wirta et al. (2016) vs. Basset et al. (2012)). Furthermore, species numbers to date mainly include the above-ground species, whereas the addition of species living below ground will further accentuate the dominance of arthropods and other invertebrate taxa. We estimate an additional hundred species contributed by mites and Collembola (Sørensen et al. 2006; Wirta et al. 2016), while enchytraeids, nematodes, and protozoa remain to be elucidated. Thus, understanding the structure of the overall web does depend on resolving even its smallest taxa—and attempts at doing so have revealed just how central in the interaction web they are (Roslin et al. 2013; Wirta et al. 2014, 2015a, 2016).

That the methods used to resolve the web will affect our impression of its structure is shown by a simple comparison: where describing associations between the main arthropod herbivores (lepidopteran caterpillars) and their enemies (parasitoid wasps and flies) by traditional rearing of larvae makes the Zackenberg food web appear as the least linked on the globe, the application of molecular techniques depicts it as the most highly linked (Wirta et al. 2014). Thus, the application of molecular tools does not only add detail to former descriptions of biotic interactions—it fundamentally changes them.

**Fig. 3:**
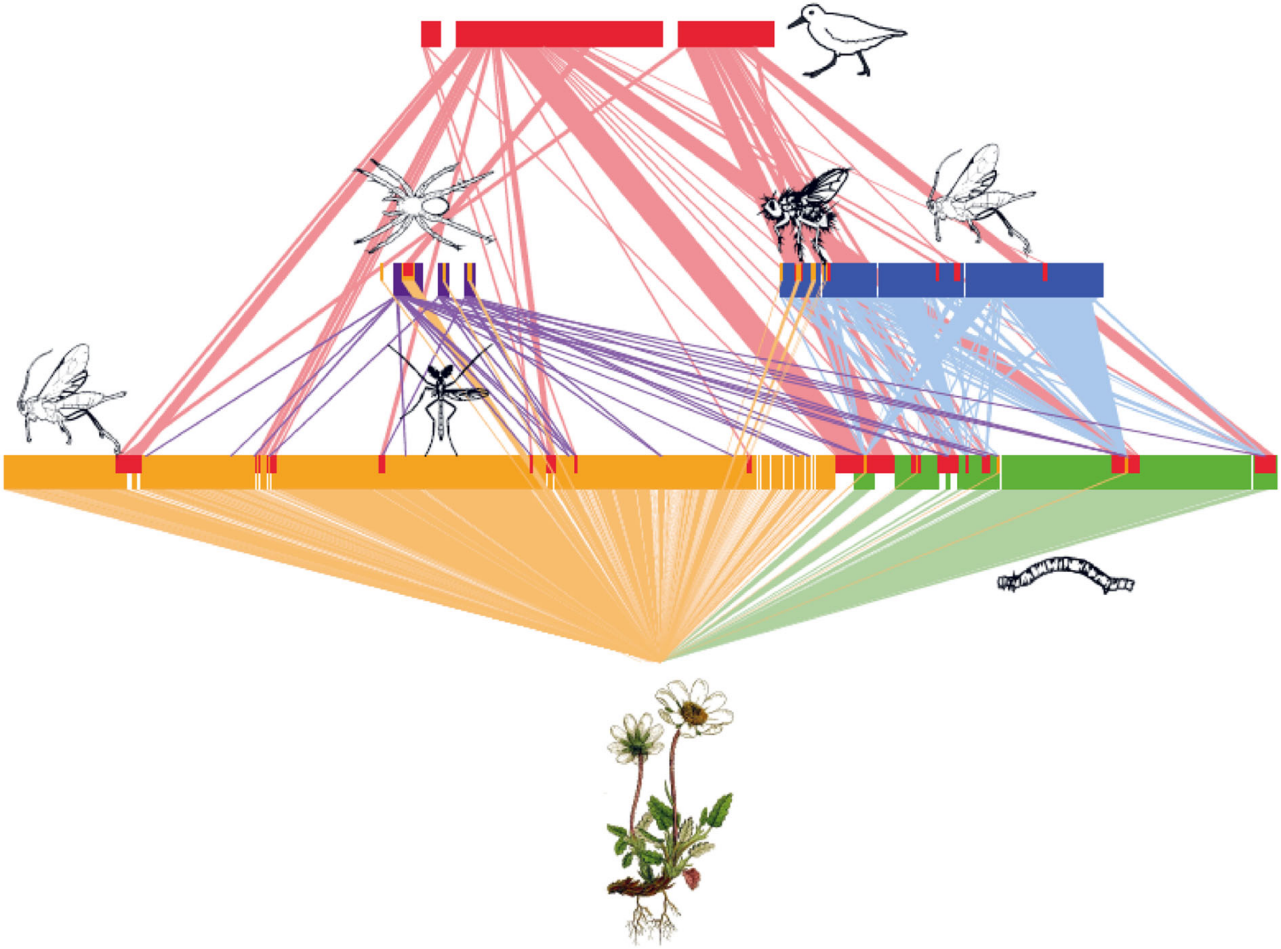
To reveal the full complexity of biotic interactions in the high arctic food web of Zackenberg, we show a quantitative representation of ecological interactions involving a single plant taxon, *Dryas octopetala* × *integrifolia*. The interactions depicted involve both antagonistic ones (*green* ^(1)^, *blue* ^(2)^, *purple* ^(3)^, and *red* ^(4)^ connectors) and mutualistic ones (*yellow* ^5^ connectors). Each *block* represents one species at a trophic level. Note that for practical reasons, the information used to quantify interaction strength varies between interaction types. (1) *Green blocks* represent Lepidopteran larvae (herbivores) found in visual searches conducted from 2009 to 2012. Only individuals found actively feeding are included here, with the widths of the *blocks* representing the numbers of individuals detected (extracted from Roslin et al. 2013). The widths of the *light green connectors* show the proportion of each herbivore taxa found feeding on *Dryas*, i.e. the relative dependence of this herbivore taxon on *Dryas*. (2) *Blue blocks* represent parasitoid species attacking the lepidopteran herbivores feeding on *Dryas* (extracted from Wirta et al. 2014). Here, the widths of the *blocks* represent the total number of interactions in which the species was involved, as detected with three different methods (MAPL-AP, MAPL-LH, and rearing; see Wirta et al. 2014). The widths of the *light blue connectors* represent the numbers of feeding events involving each herbivore. (3) *Purple blocks* represent three spider species (extracted from Wirta et al. 2014). The widths of these *blocks* represent the total numbers of feeding events involving each species, as identified with CO1 DNA barcodes, with connector width proportional to the specific predator-by-prey interaction. (4) *Red blocks* represent feeding interactions involving three bird species studied by Wirta et al. (2015a). *Blocks on the upper level* show the total numbers of feeding events detected for each bird species, and *blocks on the two lower levels* represent the total number of interactions involving each *Dryas*-affiliated prey taxon. The widths of the *light red connectors* represent the numbers of feeding events for each predator-by-prey combination. (5) *Yellow blocks* represent taxa visiting *Dryas* flowers (i.e. potential pollinators) as trapped by sticky flower mimics (from Tiusanen et al. 2016). Again, the widths of the *blocks* represent the numbers of individuals found, with widths scaled to 1/6 of those of the other *colours*, to accommodate all 185 taxa detected

That the structure of the interaction web in the high Arctic is much more complex than previously thought is visually demonstrated by Figs. 2 and 3 (as based on multiple studies and methodologies). In evidence of a densely linked structure, more than 70 % of the entire arthropod fauna known from the area has also been detected among insects visiting a single plant species, *Dryas octopetala* × *integrifolia* (Fig. 3; Tiusanen et al. 2016). Moreover, the study by Roslin et al. (2013) identified major potential for indirect effects travelling both top-down (through shared predators) and bottom-up (through shared host plants) in this system. This depiction of the arctic interaction web comes with profound implications for how it may react to ongoing change, and thus ultimately how entire ecosystems will respond to environmental change.

**Fig. 4:**
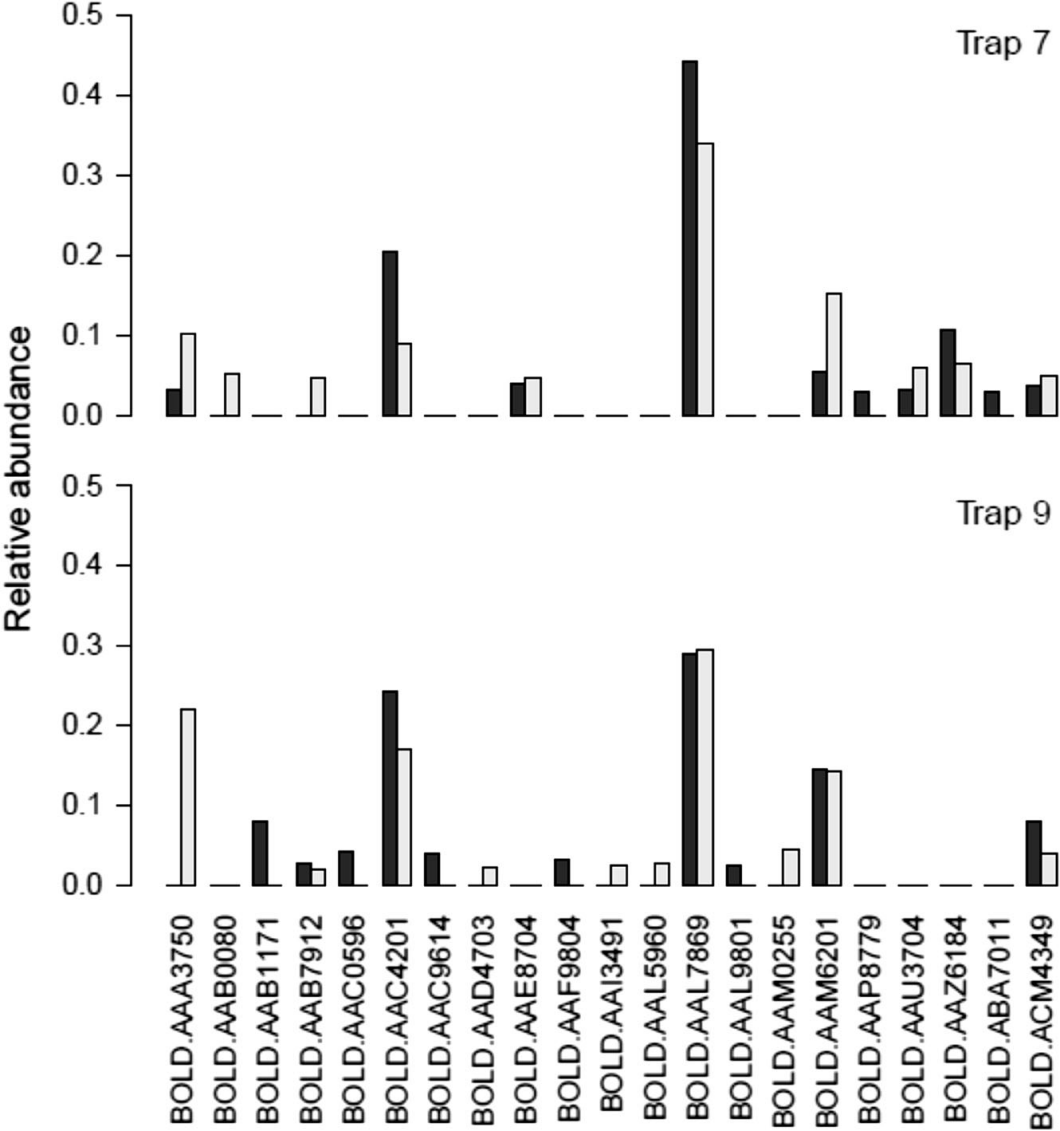
The relative abundance of arthropod species varies markedly in both space and time, here exemplified by the relative abundance of the 10 most abundant arthropod species from two malaise traps operated on two sites in 2 years at Zackenberg. For trap 7, *grey bars* indicate the year 2012 and *black bars* the year 2014, while for trap 9, *grey bars* indicate year 2013 and *black bars* year 2014. Species identities are given by their Barcode of Life (BOLD) reference code (see Wirta et al. 2016)

That the structure of the interaction web may be highly variable in space and time has been demonstrated by previous studies (Olesen et al. 2008; Rasmussen et al. 2013; Wirta et al. 2016). As an example, the fauna (and biomass) is dominated by only a few species (Wirta et al. 2016), but the abundance of these species varies dramatically between both sites and years. While the identity of the single most abundant species remained the same among two sites and years, both the identity and abundance of the nine next-most abundant taxa varied in both space and time (Fig. 4). This stresses that understanding the dynamics of individual species in this system and how these dynamics link to influences from (or to) other parts of the interaction web is a key priority for understanding how high arctic ecosystems work.

## Taxon-specific phenological responses to change

Changes in the phenology of plants and animals represent some of the most sensitive biological responses to climate change (Körner and Basler 2010; Thackeray et al. 2016), and such changes have been reported worldwide (Steltzer and Post 2009). When examined at Zackenberg, changes in phenology across a wide selection of plants and animals (Høye et al. 2007) proved much stronger than similar estimates from lower latitudes (Parmesan 2007). Strong shifts in phenology may have consequences for biotic interactions, and phenological mismatch, that is the uncoupling of interactions among individuals (conspecifics, competitors, mutualists, or individuals representing consumers and resources) in time (Miller-Rushing et al. 2010) or space (Schweiger et al. 2012). Trophic matches or mismatches between interacting species have been identified across many ecosystems and taxa (Burthe et al. 2012; Kerby and Post 2013; Thackeray et al. 2013). In arctic communities, so densely linked by biotic interactions (Fig. 2), phenological shifts among interacting species may result in functional disruption (Schmidt et al. 2016), although even strong trophic mismatches may remain without consequences (Reneerkens et al. 2016).

At Zackenberg, variation in the rate of phenological change among plants and arthropods is most often greater among plots monitoring the phenology of a given species of plants or family of arthropods than between plants and arthropods (Høye et al. 2007). Family-level taxonomic resolution of phenological time series on arthropods may however mask changes at the species level (Wirta et al. 2016). To assess the species-specific phenological responses, we have quantified the onset, peak, and end of the flight time of two abundant species of butterflies at Zackenberg. We found that the phenology of the arctic fritillary *Boloria chariclea* is advancing while the northern clouded yellow *Colias hecla* is not (Høye et al. 2014). The arctic fritillary is thus more accurately tracking changes in the timing and duration of the flowering season than the northern clouded yellow. Moreover, we found indications that the flight seasons for the butterflies were shorter in years with shorter overlap between the flowering season and butterfly flight periods (Høye et al. 2014). This adds to the more general point that phenological mismatch (or match) cannot be fully assessed by comparing relative changes in the timing of one metric of the seasonal timing of events like first flowering dates. Rather, such studies need to take the whole sequence of onset, peak, and end of the event into consideration (Post et al. 2008; Steltzer and Post 2009). At Zackenberg, we have been able to link reduced durations of flowering season to declining abundances of key flower visitors, Chironomids and Muscid flies (Høye et al. 2013) and to point to an impending community-wide functional disruption of plants and pollinators (Schmidt et al. 2016).

Differences in the timing of flowering can arise because relevant abiotic drivers are changing at different rates or because of limits to the phenotypic plasticity. Limits to phenotypic plasticity would become evident as non-linear responses to changes in underlying abiotic drivers, but there is only limited evidence of this phenomenon for plant species at Zackenberg (Iler et al. 2013). These findings suggest that in particular, the increasing temperatures but also the advancement of snow melting are driving the community-wide changes in the duration of the flowering and pollinator flight seasons, and thus ultimately the temporal overlap between the two (Høye et al. 2013; Schmidt et al. 2016).

To fully understand the importance of phenological changes, temporal (mis)matches etcetera at the community level, we need not only to resolve the interaction web, but also to be able to determine the functional importance of interacting species (Schmidt et al. 2016). Quantifying the strength of the interspecific interactions remains a major challenge.

## Herbivory—more than just removing biomass

Vegetation plays a dominant role in most interaction webs, and the consumption of plant biomass by herbivores is a central process in all ecosystems (e.g. Van der Wal 2006; Hempson et al. 2015). Due to the central role of vegetation in the interaction web, climate-induced changes in vegetation composition and biomass (Myers-Smith et al. 2011; Elmendorf et al. 2012) may change the way plants and herbivores interact, and ultimately affect the structure and functioning of the tundra ecosystem (Legagneux et al. 2014).

The only large-bodied herbivore in Northeast Greenland is the muskox *Ovibos moschatus*. At Zackenberg, the abundance of muskoxen is high compared to other arctic sites (Schmidt et al. 2015), and we have therefore examined the potential effects of their grazing activities in particular detail. During summer, muskoxen feed extensively in the productive fen areas dominated by graminoids (Kristensen et al. 2011). The fraction of the available plant biomass consumed by muskoxen in summer is, however, very low (less than 1 %; Mosbacher et al. 2016). Hence, quantitatively, muskox herbivory in summer at Zackenberg can almost be neglected. Whether this is true also during the long arctic winter is currently unknown. However, given that muskoxen rely mainly on fat depots for winter survival and reproduction (Adamczewski et al. 1997), impacts of muskox herbivory in winter are likely to be minor at the landscape scale. Nonetheless, previous studies have shown that even low-intensity muskox herbivory in tundra ecosystems may have profound effects on the plant species and communities (e.g. Tolvanen et al. 2002). Furthermore, muskoxen may be capable of counteracting climate-induced changes in the vegetation (Post and Pedersen 2008), thus impacting the stability of plant communities (Post 2013). In addition to the grazing impacts, muskoxen may affect plant communities by impacting nutrient turnover and relocation (Mosbacher et al. 2016) and by impacting the vegetation, and especially mosses, through trampling (Falk et al. 2015). In particular, suppression of the moss layer by trampling in tundra ecosystems may have disproportionate effects, as mosses influence important factors such as soil temperature and moisture (Hobbie et al. 2000; Gornall et al. 2009). Indeed, Gornall et al. (2009) concluded that herbivore impacts on the moss layer are the key to understand the response of tundra ecosystems to warming and grazing.

Even though the impact of the other vertebrate herbivores at Zackenberg (collared lemmings, arctic hares, ptarmigans, and geese) (Berg et al. 2008) have not been studied in detail, their generally low abundances suggest their impact on the vegetation is likely to be only local.

In contrast to vertebrate herbivory, invertebrate herbivory in tundra ecosystems has often been overlooked by the research community (Haukioja 1981). This may be partly due to the fact that invertebrate herbivores generally remove less biomass and have smaller impact on plants as compared to their larger vertebrate counterparts (Crawley 1989; Kotanen and Rosenthal 2000). Studies at Zackenberg have revealed that the consumption of plants by invertebrate herbivores is indeed low (Roslin et al. 2013), but still of the same magnitude as the consumption by the muskoxen (Mosbacher et al. 2016). The life cycles and metabolism of invertebrates are, however, more responsive than those of vertebrates to increasing arctic temperatures (O’Connor 2009; Rall et al. 2010; Amarasekare and Sifuentes 2012). For instance, the abundance of the arctic aphid *Acyrthosiphon svalbardicum* increased markedly in response to warming (Hodkinson et al. 1998). The only experimental study on invertebrate herbivores conducted at Zackenberg, however, failed to detect any changes in eriophyoid gall mite (Acari; superfamily Eriophyoidea) abundance in response to altered environmental conditions (Mosbacher et al. 2013). Nonetheless, other studies have shown that when subject to warming, the general level of invertebrate herbivory may increase significantly (Richardson et al. 2002). In fact, it has been suggested that herbivory is already increasing concomitantly with the ongoing global warming (Tylianakis et al. 2008). As the Arctic warms, outbreaks of herbivorous insects may also become more frequent, thus resembling the situation found in the Subarctic and low Arctic today (Jepsen et al. 2008).

## Tolerance towards loss, gain, or change in the relative abundance of species

By definition, the structure of the interaction webs changes when new species enter or leave the communities. Model studies have assessed the impacts of species loss from interaction webs (e.g. Memmott et al. 2004), but unravelling the impacts of changes in the structure of real interaction webs is inherently difficult (but see Brosi and Briggs 2013). Furthermore, changes in the relative abundance of species (both resources and consumers) usually impact the functioning and dynamics of the communities without necessarily changing the overall structure (i.e. the number and identity of composing species) of interaction webs.

The lemming–predator community in Northeast Greenland serves as an illustrative example of how quantitative changes in an interaction web result in changes in the abundance of key species under natural conditions and, in turn, in qualitative changes of the interaction web, such as local extinctions (e.g. Gilg et al. 2009). In the tundra ecosystem, lemmings and voles constitute the main food base for a number of vertebrate predators (Gilg et al. 2006; Schmidt et al. 2008, 2012), and in Greenland, only one small rodent species is found, the collared lemming *Dicrostonyx groenlandicus*. Until the year 2000, the populations exhibited classical cyclic, large-amplitude fluctuations, but since then, the cyclic pattern has disappeared, and densities have remained at a low, relatively stable level (Gilg et al. 2009; Schmidt et al. 2012).

As the reproductive success of most terrestrial vertebrate predators in the Arctic depends on high lemming abundances, the lemming collapse has resulted in declining reproductive outputs in trophically linked species. However, even in this simple vertebrate interaction web with just a few vertebrate predator species and one major prey species (Gilg et al. 2003), understanding the full extent of a decline or loss of a key prey species is challenging. The impacts of declining prey on the predator community depend on predator species as well as local availability of alternative food sources (Schmidt et al. 2012). Hence, while the snowy owl *Bubo scandiacus* almost completely ceased reproducing, reproduction of the Arctic fox *Vulpes lagopus* only declined moderately, and the long-tailed skua *Stercorarius longicaudus* exhibited an intermediate decline. These differences are due to the varying degree of dietary specialization of the predators, and thus the availability of alternative prey. Hence, the Arctic fox suffers the least due to its flexible diet (Ehrich et al. 2015), and the number and strength of links to the Arctic fox (Fig. 2f), thus buffers the immediate negative impacts of reduced lemming prey. In the more specialized long-tailed skua, the reproductive output also declined, but the large fraction of potential breeders that remain non-territorial until territories become available may delay the negative impact of the lemming collapse on the long-tailed skua breeding population (Barraquand et al. 2014). Thus, the duration of the lemming collapse is crucial for the severity for the predator guild. Additional complexity to our understanding of the effects of species loss comes from the fact that the geographical extent of the collapse also impacts the predator species differently due to their varying degree of mobility and site fidelity (Barraquand et al. 2014; Therrien et al. 2014).

The direct links between lemmings and their predators in the above case may, however, be a notable exception for the Arctic. In fact, the majority of interaction sub-webs depicted in Fig. 2 are characterized by a dense linkage structure (i.e. high connectivity), and thus dominated by generalist species (Wirta et al. 2015a). Indeed, the pattern of high generalism also extends to the plant–pollinator web, where individual pollinators tend to visit a large fraction of plant species available (Fig. 2) (Rasmussen et al. 2013). The large number of shared predators, prey, and food plant species observed in the interaction webs at Zackenberg potentially allows for environmental changes to cascade onto the entire interaction web through indirect interactions. For instance, Mortensen et al. (2016) showed how climate impacts may propagate through the tri-trophic system of plants–arthropods–shorebirds at Zackenberg by means of direct and indirect effects, impacting the phenology and performance at the various trophic levels. Such cascading effects, affecting entire interaction webs, may ultimately affect the way the tundra ecosystem is structured and the way it functions (Ims et al. 2008; Legagneux et al. 2014). From an ecosystem perspective, the high degree of generalism observed in the webs at Zackenberg (Fig. 2) may however also cause resilience/resistance (as impacts onto the interaction web are diluted through its many channels) (Strong 1992; Bartomeus et al. 2013). An illustration of this was provided by the experimental study by Visakorpi et al. (2015). While predicting pronounced trophic cascades in the presumptively simple food webs of the Arctic, they expected that the removal of predators would enhance herbivory by increasing the number of herbivores, and that an increase in predator species would come with the opposite effect. However, this proved not to be the case, as no detectable effects emerged. Thus, this part of the interaction web seems rather robust against cascading effects originating from changes in the densities of single species—a pattern which the authors attributed to the effects of elevated predation pressure being diluted through multiple parallel channels in the complex food web. Similarly, in the lemming example above, the Arctic fox in particular has multiple trophic links to alternative prey species (e.g. muskox carcasses, fish, egg, and young of ground-nesting birds), and the decline in its lemming prey could therefore have negative consequences for the alternative prey species through increased predation rates (Summers et al. 1998; Aharon-Rotman et al. 2014).

## Arctic birds depend on their arthropod prey

Many migratory bird species depend to a large extent on arthropods as a food source during the period of reproduction on the arctic tundra (Meltofte et al. 2007b). Arthropods are the only food source for shorebirds and snow buntings *Plectrophenax nivalis*, whereas long-tailed skuas eat arthropods as an alternative (and additional) food source to lemmings (Meltofte and Høye 2007). Molecular techniques revealed that insectivorous birds in Zackenberg are generalist predators feeding on nearly all available arthropod species (Wirta et al. 2015a). Shorebirds rely heavily on local arthropods for the production of their eggs (Klaassen et al. 2001), and early spring arthropod abundance is thus an important determinant of the date of egg laying (Meltofte et al. 2007a). Daily fluctuations in ambient temperature and arthropod abundance determine incubation schedules in adult uniparental shorebirds (Reneerkens et al. 2011) and time budgets of precocial shorebird chicks (Krijgsveld et al. 2003). While it is clear that in the tundra biome, birds depend on the arthropods, the impact of bird predation on the arctic arthropod community has not yet been thoroughly evaluated (e.g. Appendix B in Visakorpi et al. 2015).

Especially in the Arctic, arthropod phenology has advanced much faster than that of their shorebird predators in response to a warming climate (Høye et al. 2007; Tulp and Schekkerman 2008). Given the temporally extended peak of arthropod abundance in Zackenberg (Høye and Forchhammer 2008) compared with other arctic regions (Tulp and Schekkerman 2008; Bolduc et al. 2013), current phenological mismatches will not necessarily result in negative fitness consequences for the avian predators. Effects may be limited, as long as arthropod prey abundance exceeds a minimal threshold for sufficient chick growth for a long time after the absolute annual peak in food abundance (Durant et al. 2005). Indeed, the growth of sanderling *Calidris alba* chicks in Zackenberg was not affected by the extent of the phenological mismatch with the date of the seasonal maximum abundance of their arthropod prey, but generally chicks grew better when the arthropod peaks were broad and high (Reneerkens et al. 2016). Different arthropod groups advance at different paces in response to climate warming (Høye and Forchhammer 2008), which may also affect the quality of shorebirds’ diet (cf. Razeng and Watson 2015). To understand how the reproductive success of birds is affected by changing arthropod resources, we still need better quantification of the proportions of the various arthropods in the diets of arctic insectivorous birds. We also need better-resolved descriptions of spatial and temporal variation in arthropod abundance in relation to the local movements of the birds and to get a better understanding of the relative importance of bottom-up (via arthropods) or top-down (via predation on bird eggs and chicks) effects on the reproductive output of birds (Reneerkens et al. 2016). 

## Cross-boundary interactions

So far, our research, and thus this review, has focused on the terrestrial interaction web at Zackenberg. However, most interaction webs are affected by cross-boundary exchange of resources from one ecosystem to another. In the arctic terrestrial interaction web such allochthonous resources come from both the marine and the limnic environments. Resources from the marine environment are for instance transferred to the terrestrial ecosystem by many of the terrestrial predators (Therrien et al. 2011; Tarroux et al. 2012; Gilg et al. 2013), and in some cases herbivores, too, make extensive use of marine resources outside the summer season (Hansen and Aanes 2012). The terrestrial system receives input from the limnic ecosystem, through the consumption of freshwater midges by terrestrial spiders (Gratton et al. 2008). In less pristine areas, human subsidies may also be an important factor affecting the biotic interactions (e.g. Julien et al. 2014). Migratory animals are another (extreme) example of how ecosystems, even over vast geographical distances, may be reciprocally linked (Bauer and Hoye 2014). Our understanding of the importance of such cross-boundary interactions for the structure and function of the interaction web at Zackenberg is currently scant, and is mainly restricted to the most obvious ones, e.g. marine input to diets of terrestrial predators (Ehrich et al. 2015) and no human subsidies. Hence, in developing our understanding of the terrestrial interaction web at Zackenberg and elsewhere in the Arctic, we need to improve our understanding of how interaction webs link locally across barriers and globally across latitudes, and understand how these cross-boundary interactions vary over time and with changes in for instance climate.

## Conclusions and perspectives

The dissection of the interaction web at Zackenberg serves to expose the true complexity of the arctic interaction webs. The complexity unravelled to date is likely to increase even further. So far, we have only just started to map out the interaction web, focusing mainly on the above-ground interaction webs, and mainly during the summer period. As we in the future successfully map the more subtle (e.g. Kutz et al. 2004; Meyling et al. 2012), the infrequent interactions (e.g. Chevallier et al. 2016) and as we include more compartments, such as the below-ground interactions, the interaction web and our perception of it will change accordingly. Adding more species into the interaction web will inevitably increase the complexity, merely because of the increasing number of (potential) linkages. On the other hand, adding information on the abundance of the individual species as well as the strength of the individual linkages in the interaction web, i.e. taking a more functional view on the interaction web, may result in what can be seen as a less complicated interaction web, as the various species in the web are found in varying numbers and come with varying functional importance (see e.g. Schmidt et al. 2016). Still, even weak links may be very important in interaction webs (Rooney et al. 2006). Deciphering the interaction web outside the summer season is probably not going to change our general perception of the web, as most biotic interactions takes place outside the snow-covered period. Nonetheless, winter processes and events (Bokhorst et al. 2016) may indeed be pivotal for the interaction web and set the scene for what we observe in summer and must therefore be considered.

Of the patterns emerging from our efforts to reveal the interaction web at Zackenberg, some are indeed likely to be site-specific, while others will apply throughout the Arctic. We hope that the emerging view on the Zackenberg interaction web provided here (Figs. 2, 3) will stimulate a new view on arctic food web ecology in general, guide hypotheses, and aid the detection of knowledge gaps.

While mapping out the interaction web at Zackenberg and elsewhere, one must keep in mind that interaction webs are highly dynamic, and that species abundances may change and that new species may enter the web. The view emerging from our synthesis provides suggestions of how species turnover and changes in the relative abundance of individual species may affect arctic interaction webs. As suggested above, arctic interaction webs may be characterized both by a highly connected structure (essentially passing on influences among interacting species) and generalism (allowing flexible shifts along resources when needed). Currently, most new species appearing in the Arctic are the result of range expansions, but some appear due to human action (Bennett et al. 2015). The establishment of new species in the arctic ecosystems (Killengreen et al. 2007; Nielsen and Wall 2013; Alsos et al. 2015; Coulson 2015), and thus the formation of new, currently unknown, biotic interactions is a major challenge for our understanding of the arctic ecosystems of tomorrow (Walther et al. 2009). Although Zackenberg lies rather isolated on the east coast of Greenland, new species will eventually arrive and will likely enter the interaction webs there. So far, we have documented a few new species in the tundra ecosystem at Zackenberg (e.g. the Greenland ladybird *Coccinella transversoguttata* (Böcher 2009), and the Lapland bunting *Calcarius lapponicus*).

While having the capacity to change the arctic interaction web profoundly, the realized result of such new species invading an existing web depends on both the structure of the existing web and the strength of the interactions that the newcomer is able to build. Given the complexity of the webs emerging here, predicting the outcome of species invasions and extinction is difficult, and urgently calls for modelling of the interaction webs, and for empirical tests of model predictions. Both climate-induced changes in the vegetation (e.g. Elmendorf et al. 2012) and the establishment of new species in the Arctic are likely scenarios of the Arctic of tomorrow, resulting in communities and climates that are different from what we know today (Williams and Jackson 2007; CAFF 2013). Only continued long-term monitoring (Lindenmayer et al. 2010), coupled with research dedicated to map out the interaction web, will allow us to both keep track of and understand these pivotal changes.
